# Rural Urban Disparities in Geriatric Care Resources and Usage

**DOI:** 10.1093/geroni/igaf122.1231

**Published:** 2025-12-31

**Authors:** Orna Intrator, Sara Elgayar, Porooshat Dadgostar, Lynette Kelley, Emily Fanzosa, William Hung

**Affiliations:** James J Peters VA Medical Center, New York, New York, United States; The University of Alabama at Birmingham, Birmingham, Alabama, United States; University of Rochester, Rochester, New York, United States; Veterans Health Administration, Aurora, Colorado, United States; James J Peters VA Medical Center, New York, New York, United States; James J Peters VA Medical Center, New York, New York, United States

## Abstract

Disparities in availability of geriatric care may lead to limited access of geriatric care for older adults, especially those residing in rural areas. Mapping locations of geriatric resources and needs will identify incongruities between supply and demand. Through the use of data from multiple sources including Medicare data and from the Veteran Healthcare System, we used an annual repeated cohort approach of older adults in the Veteran Healthcare system using VA and Medicare data to identify usage of geriatric care services across health systems. Older adults are categorized by their geographic location using RUCA codes matched to their residential zip-code, categorized into urban, rural and highly rural areas. We compare and contrast the characteristics of older adults who utilize geriatric care services using complexity criteria previously established including risk of hospitalization or mortality, high cost utilization, and long term institutionalization, frailty, and specific geriatric conditions including dementia and falls. We also identify the distribution of care providers in geriatric specialties using the National Provider Identifier comparing rates in rural and urban areas.

